# Differential responses to the combination of navitoclax and venetoclax with doxorubicin in murine models of triple negative breast cancer

**DOI:** 10.3389/fcell.2026.1661424

**Published:** 2026-02-05

**Authors:** Homood M. As Sobeai, Abdulrahman M. Alanazi, Faisal Alotaibi, Ali Alhoshani, Khalid Alhazzani, Mashal M. Almutairi, Sultan Almudimeegh, Basel K. Al-Ramadi, Eesha Chakraborty, Tareq Saleh, David A. Gewirtz, Hisashi Harada, Moureq R. Alotaibi

**Affiliations:** 1 Department of Pharmacology and Toxicology, College of Pharmacy, King Saud University, Riyadh, Saudi Arabia; 2 Pharmaceutical Care Division, King Faisal Specialist Hospital and Research Centre, Madinah, Saudi Arabia; 3 Inpatient Pharmacy Department, King Fahad Medical City, Riyadh, Saudi Arabia; 4 Department of Medical Microbiology and Immunology, College of Medicine and Health Sciences, United Arab Emirates University, Al Ain, United Arab Emirates; 5 Department of Pharmacology and Toxicology, School of Medicine, Virginia Commonwealth University, Richmond, VA, United States; 6 Massey Comprehensive Cancer Center, Virginia Commonwealth University, Richmond, VA, United States; 7 Department of Pharmacology & Therapeutics, College of Medicine and Health Sciences, Arabian Gulf University, Manama, Bahrain; 8 Department of Pharmacology and Public Health, Faculty of Medicine, The Hashemite University, Zarqa, Jordan; 9 School of Dentistry, Philips Institute for Oral Health Research, Virginia Commonwealth University, Richmond, VA, United States

**Keywords:** apoptosis, breast cancer, doxorubicin, navitoclax, senescence, venetoclax

## Abstract

**Introduction:**

Therapy-induced Senescence (TIS) can potentially influence breast cancer treatment outcomes, in part by contributing to disease recurrence; hence, the utilization of senescence-eliminating agents (i.e., senolytics) is considered as a possible adjuvant to chemoradiation. However, one of the most effective senolytic agents, navitoclax (ABT-263), is limited by its associated toxicities of thrombocytopenia and neutropenia. In contrast, venetoclax (ABT-199), which is currently standard of care in CLL and AML, is of less senolytic potential. Moreover, a comparison between their combinational effect with standard chemotherapy in animal models of breast cancer is not widely explored. This study compared the senolytic potential of the two BH3 mimetics in combination with doxorubicin in two models of triple-negative breast cancer (4T1 and E0771 cells).

**Methods:**

Senescence was cytochemically confirmed via Senescence-associated β-galactosidase upregulation (and quantified by flow cytometry), *CDKN1A* induction, and the senescence-associated secretory phenotype (SASP) expression (using qRT-PCR). Cell viability and the percentage of apoptotic cells were determined using MTT and Annexin V/7AAD assays, respectively.

**Results:**

Both navitoclax and venetoclax were effective as apparent senolytics in the E0771 cells. In contrast, only navitoclax was effective against the 4T1 cells. The *in vitro* findings in E0771 cells were validated through studies conducted in vivo in immunocompetent mice implanted with E0771-derived tumors where both drugs reduced tumor progression and shifted cells to apoptosis in sequential combination with doxorubicin.

**Conclusions:**

These findings suggest that administration of venetoclax has the potential to enhance suppression of doxorubicin-exposed cancer cells, and that it may have potential as that of Bcl-xL-targeting agents. However, given the variable outcomes in the two triple-negative breast tumor cell lines, it becomes incumbent to identify the factors that confer susceptibility to Bcl- 2 targeting agents in anticipation of their potential utilization in the clinic for combination therapy in solid tumors.

## 1 Introduction

Breast cancer is the most frequently diagnosed cancer among women worldwide and is a leading cause of cancer-related mortality (Bazarbashi et al., 2017; Althubiti and Eldein, 2018; Bray et al., 2024; Siegel et al., 2024). Relapse, often due to treatment failure, significantly contributes to breast cancer mortality (Ramos and Bentires-Alj, 2015; Colleoni et al., 2016). Therefore, identifying breast cancer patients at high risk of disease recurrence and developing more effective therapeutic strategies are crucial for improving overall survival. Resistance to chemotherapeutic agents is a central problem in cancer treatment (Boldrini, 2000; Smith et al., 2019). Tumors often respond to treatment initially but can develop resistance through diverse molecular and cellular mechanisms (Rebucci and Michiels, 2013; Pan et al., 2016; Nedeljković and Damjanović, 2019; Bukowski et al., 2020; Davodabadi et al., 2023). These mechanisms include the treatment-induced selection of aggressive tumor cell subpopulations that survive chemotherapeutic treatment (Venkatesan et al., 2017; Shnaider et al., 2020; Shi et al., 2023). Addressing how cancer cells evade chemotherapy is essential for improving treatment efficacy and patient outcomes.

Therapy-Induced Senescence (TIS) plays a critical role in the tumor cell response to anticancer treatments, both *in vitro* and *in vivo*, and is considered a hallmark of cancer (Saleh et al., 2020a; Saleh et al., 2023; Hanahan, 2022). While TIS can initially act as a barrier to tumor growth, it is increasingly recognized as a double-edged sword, reflective of an unfavorable outcome of cancer therapy (DeLuca and Saleh, 2023). A major concern is that some senescent tumor cells can escape the growth arrest induced by senescence, evolving into more aggressive, stem cell-like variants (Saleh, 2024) that contribute to the evasion of immunosurveillance, leading to tumor dormancy and ultimately, disease recurrence (Demaria et al., 2017; Yang et al., 2017; Milanovic et al., 2018; Muñoz et al., 2019; Saleh et al., 2019b; Saleh et al., 2019a; Duy et al., 2021; Saleh and Gewirtz, 2022; Shahbandi et al., 2022). Consequently, ongoing research efforts have focused on developing strategies to target and eliminate senescent tumor cells, with the goal of improving treatment efficacy (Wang et al., 2017; Short et al., 2019).

Senotherapeutics, a class of drugs targeting senescent cells, are emerging as potential solutions (Bousset and Gil, 2022). These include senolytics, which selectively induce apoptosis in senescent cells; senomorphics, which modify the senescence-associated secretory phenotype (SASP); and senostatics, which maintain growth arrest and prevent escape from senescence (Khalil et al., 2023). However, discussion is still ongoing on their senescence-selective synergistic effects when combined with senescence-inducing standard chemotherapy. Moreover, a high degree of variability in the efficacy of these various compounds has been reported in cancer models. One reason may be that tumor-bearing animal models utilized to screen for or test different senotherapeutics have predominantly relied on xenografts implanted into immunodeficient hosts. This approach carries limitations, especially in light of the complex and well-documented ways in which the SASP interacts with the immune system. Notably, during the initial 1–3 weeks following the development of TIS, the SASP frequently triggers immune responses that contribute to tumor elimination. This phenomenon has been observed in models of breast cancer (driven by CDK4/6 inhibitors), hepatocellular carcinoma (via p53-induced senescence), and in early-stage liver lesions (induced by ionizing radiation or KRAS^G12V^ mutations) (Goel et al., 2017; Sturmlechner et al., 2021). However, after a few weeks, this immune-mediated suppression of tumor growth tends to subside, allowing tumors to potentially regrow (Chibaya et al., 2022; Liu et al., 2024; Bajtai et al., 2025). To fully understand the optimal utilization of senescence-inducing (senogenic) and senolytic therapies, more comprehensive studies in immunocompetent animal models are required.

Both navitoclax and venetoclax are notable examples of senolytics with some clinical utility as anticancer agents against hematological malignancies, including chronic lymphocytic leukemia (CLL) and acute myelogenous leukemia (AML). Venetoclax is a BH3-mimetic drug that targets only Bcl-2, thereby differing in its selectivity and toxicity profiles from navitoclax. Navitoclax inhibits both Bcl-2 and Bcl-xL, making it a potent senolytic, particularly against TIS tumor cells (Wang et al., 2017). However, the inhibition of Bcl-xL leads to significant adverse effects, most notably thrombocytopenia, which is often dose-limiting and restricts its clinical use (Tse et al., 2008). Venetoclax, on the other hand, selectively inhibits Bcl-2 and spares Bcl-xL, resulting in a more favorable toxicity profile with a lower risk of thrombocytopenia (Inoue-Yamauchi et al., 2017). Although venetoclax is now standard-of-care for the treatment of Bcl-2-dependent cancers, specifically CLL and AML, its senolytic efficacy against TIS tumor cells is generally lower compared to navitoclax due to its lack of Bcl-xL inhibition (Saleh et al., 2025; Rahman et al., 2022; Jochems et al., 2024). Unfortunately, there is a dearth of information relating to the senolytic potential of venetoclax in solid tumor models. In this work, we evaluated the combination effects of navitoclax and venetoclax with doxorubicin in two murine triple-negative breast cancer models *in vitro* and in one model in immunocompetent mice.

## 2 Materials and methods

### 2.1 Cell lines and drug treatment

4T1 and E0771 murine cell lines were purchased from American Type Culture Collection (ATCC, Manassas, Virginia, United States). Selecting these cell lines allowed for the examination of senescence induction and elimination in immunocompetent mouse models. Cells were cultured and maintained in Dulbecco’s Modified Eagle Medium (DMEM) supplemented with 10% Fetal bovine serum and 1% penicillin/streptomycin, all sourced from Gibco (Waltham, Massachusetts, United States). Doxorubicin hydrochloride, venetoclax, and navitoclax, were obtained from Selleckchem (Houston, Texas, United States)**.** The drugs were dissolved in dimethyl sulfoxide (DMSO) and handled in the dark at the desired quantities to achieve a final DMSO concentration in media (0.1%) or less.

### 2.2 Experimental design

**FIGURE 1:**
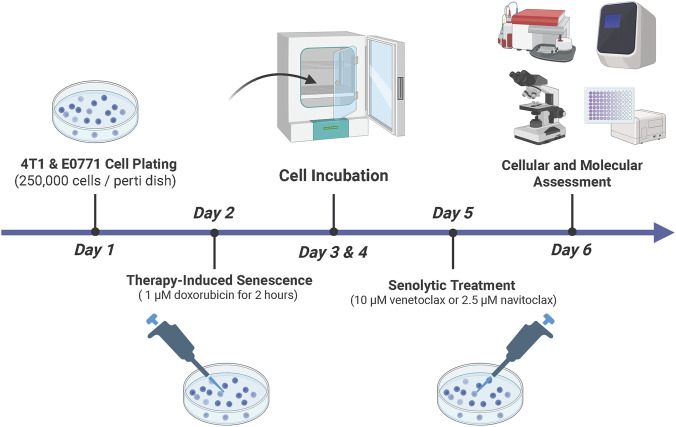
Experimental timeline. A total of 250,000 4T1 and E0771 cells were seeded and, on the following day, pulse-treated with 1 µM doxorubicin for 2 h. The medium was then replaced with fresh culture medium and cells were incubated for 72 h (3 days) to allow senescence to reach its peak. Subsequently, cells were treated with either 10 µM venetoclax or 2.5 µM navitoclax for 24 h. On the final day, cells were subjected to cellular and molecular analyses.

A screening strategy was used to identify the optimal time for the combination of doxorubicin and senolytics to induce the death of therapy-induced senescent cells in the cell lines. Cells were treated based on the following experimental groups: drug-free vehicle (control), doxorubicin, venetoclax, navitoclax, and the combination therapy of either doxorubicin and venetoclax or doxorubicin and navitoclax. TIS was established by pulse-exposure of doxorubicin with a concentration of 1 µM for 2 h, as demonstrated previously (Saleh et al., 2020c). Cells were then washed with phosphate-buffered saline and fed with a fresh culture medium. Once TIS reached its peak at day 3, cells were treated with venetoclax or navitoclax. Three independent experiments were performed for all the experimental work presented in this study. The experimental timeline is illustrated in Figure 1.

### 2.3 SA-β-gal staining and C_12_-FDG quantification to evaluate senescence

Cells were seeded into 6-well plates and treated with doxorubicin as indicated above in the Section 2.2. SA-β-galactosidase staining was performed using the Senescence β-Galactosidase Staining Kit (#9860, Cell Signaling Technology, Danvers, Massachusetts, United States). Once completed, the plate was imaged under a light microscope (×100 magnification). C_12_-FDG quantification is a flow cytometry-based assay used as a surrogate to detect SA-β-gal, as reported previously (Debacq-Chainiaux et al., 2009; Softah et al., 2023). Bafilomycin A1 was added to cultured cells to increase the cellular pH to six followed by incubation of the cells with C_12_-FDG, the fluorogenic β-gal substrate (ThermoFisher Scientific, Waltham, Massachusetts, United States), which is cleaved by β-gal and emits fluorescence. C_12_-FDG positive cells were considered to be senescent while C_12_-FDG negative cells were considered to be non-senescent.

### 2.4 MTT cell viability assays

Ten thousand cells were seeded in a 96-well plate for MTT assays. Cells were treated as described in the Section 2.2. On the day of measurement, the medium was removed and replaced with 100 µL of fresh culture medium. 10 μL of the 12 mM MTT stock solution (ThermoFisher Scientific) was added to each well. The plates were incubated at 37 °C for 4 h. The medium containing MTT solution was removed, and 100 µL of isopropyl alcohol was added to dissolve purple crystalline precipitates formed inside the cells. The plates were placed on a shaker for 10 min and then measured at 570 nm using Mithras^2^ LB 943 microplate reader (Bad Wildbad, Baden-Wurttemberg, Germany) (As Sobeai et al., 2021).

### 2.5 FITC annexin V/7-AAD assay to measure apoptosis

Apoptosis was evaluated in the experimental groups using the FITC Annexin V/7-AAD assay, as reported (van Genderen et al., 2006). Briefly, cells in the experimental groups were collected and centrifuged after the treatment protocol. The medium supernatant was discarded, and the cell pellet was reconstituted in 500 µL of binding buffer containing 5 µL of annexin V-FITC and 5 µL of 7-AAD (BioLegend, San Diego, California, United States). Samples were incubated at room temperature for 15 min, shielded from light sources, and processed using Cytomics FC 500 (Beckman Coulter, Brea, California, United States).

### 2.6 RT–PCR to measure senescence canonical gene expression

The influence of the treatment protocols on the expression of key senescence-related genes such as *CDKN1A*, and *IL6* was investigated using RT-PCR. Total RNA was harvested using the mRNeasy mini kit (Qiagen, Hilden, Germany) following the manufacturer’s protocol. Total RNA quantity and purity were determined by measuring absorbance at 260 and 280 nm using NanoDrop 8,000 Spectrophotometer (ThermoFisher Scientific), respectively. Two micrograms of total RNA were converted to cDNAs using the high-capacity cDNA reverse transcription kit (ThermoFisher Scientific, Waltham, Massachusetts, United States). Then, 40 PCR cycles were performed using QuantStudio 6 Flex RT-PCR System (Applied Biosystems, Massachusetts, United States). Primers of genes of interest, *CDKN1A* (Mm00432448_m1), *IL6* (Mm00446190_m1), and *GAPDH* (ID Mm99999915_g1). Primers were purchased from ThermoFisher Scientific. Data was expressed as a fold change based on the 2^−ΔΔC^_T_ method (Hanbashi et al., 2023).

### 2.7 Relative free survival analyses based on senescence gene expression profiles

The prognostic significance of the indicated senescence-associated genes was interrogated utilizing the KMploltter database (https://www.kmplot.com) (Györffy et al., 2010; Győrffy, 2024). Relative free survival curves of breast cancer patients that underwent chemotherapy were generated (n = 1935). Median survival values in months and hazard ratios were computed. The statistical significance was analyzed using logrank. *P* < 0.05 is considered statistically significant.

### 2.8 *In vivo* evaluation of venetoclax and navitoclax senolytic efficiency against E0771-derived tumors in immunocompetent C57BL/6 mice

The impact of venetoclax or navitoclax on *in-vivo* tumor growth following doxorubicin treatment was evaluated in female immunocompetent C57BL/6 mice aged 6–8 weeks implanted with E0771 cells. 2.5 million cells were suspended in 100 µL DMEM-matrigel solution (1:1 dilution) and injected subcutaneously into the rear flank of mice. The tumor volume was assessed utilizing Vernier caliper measurements every day until the volume reached around 200 mm^3^, when the mice were randomized into four groups (n = 6 per group): mice treated with drug-free vehicle, mice treated with two intraperitoneal (IP) doxorubicin doses of 2.5 mg/kg on day 0 and day 4 after tumor detection, mice treated with four oral venetoclax or navitoclax doses of 50 mg/kg on days, 6, 8, 10, and 12, and mice treated with the combination therapy of doxorubicin and venetoclax/navitoclax based on the aforementioned dosing schedules. Tumor volumes were measured across four experimental groups every other day for 2 weeks. The animal studies were performed in accordance with the Institutional Animal Care and Use Committees in King Saud University guidelines (Ethical code reference number: KSU-SE-21–28).

### 2.9 Histopathological investigations of tumor tissues *in vivo*

Tumor nodules were excised and subsequently fixed in a 10% formalin solution for a period of 7 days. Post-fixation, the samples underwent a series of dehydration, clearing, and infiltration procedures, followed by embedding in paraffin wax. The paraffin blocks were then sectioned into 6 µm thick slices, which were dried at 65 °C. These sections were stained using hematoxylin and eosin to facilitate histological examination. Photomicrographs of the stained sections were captured using Nikon YS100 light microscope (Tokyo, Japan) at a magnification of 400X. Quantitative image analysis was performed using ImageJ software version 1.54 g (National Institutes of Health, Bethesda, Maryland, United States).

### 2.10 TUNEL assay of tissue samples in response to treatment

Apoptosis in tumor tissues from the *in vivo* experiments was assessed using the TUNEL Assay (BrdU-Red, Abcam, Cambridge, United Kingdom). Briefly, formaldehyde-fixed tissues were washed and digested with proteinase K for 5 min at room temperature. After a washing step, the samples were incubated with the DNA labeling solution for 60 min at 37 °C, washed, and subsequently treated with the antibody solution for 30 min at room temperature. The samples were then incubated with the 7-AAD/RNase A solution for another 30 min at room temperature and analyzed under a fluorescence microscope. The extent of apoptosis was determined by measuring the intensity of the BrdU-Red signal. Fluorescence emission was measured at excitation/emission wavelengths of 488/576 nm (Softah et al., 2023).

### 2.11 Statistical analysis

Data were illustrated as means ± standard errors of the means of at least three independent experiments. Statistical significance was examined utilizing one-way analysis of variance (ANOVA) followed by Tukey’s honest significance (p < 0.05). The statistical analyses were performed, and figures were generated using GraphPad Prism 10.2.3 (San Diego, California, United States).

## 3 Results

### 3.1 Doxorubicin induces senescence in 4T1 and E0771 breast cancer cells

**FIGURE 2:**
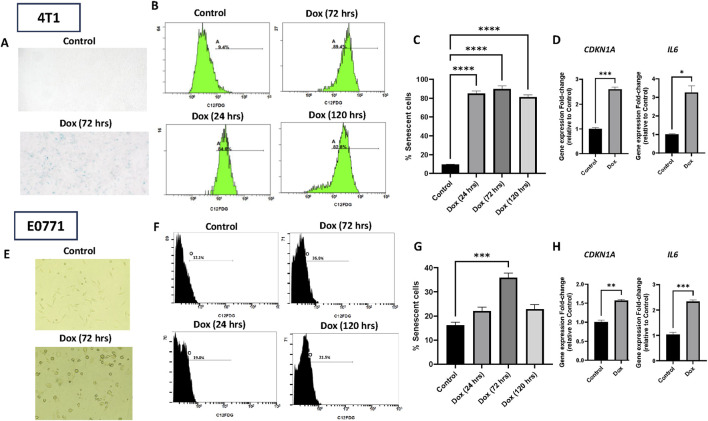
Doxorubicin induces therapy-induced senescence (TIS) in murine breast cancer cell lines. TIS was triggered in 4T1 and E0771 cells by doxorubicin (1 µM) treatment for 2 h. Cells were incubated for 1 day (gray), 3 days (dark gray), and 5 days (light gray). Drug-free controls were included in the experiments (black). β-gal staining and C_12_-FDG quantification were utilized to evaluate TIS. **(A)** Representative microscopic images of untreated and doxorubicin-treated (72 h) 4T1 cells stained with β-gal. **(B)** Representative flow cytometry charts of 4T1 C_12_-FDG positive cells in the experimental groups. **(C)** Statistical quantification of 4T1 C_12_-FDG positive cells in the experimental groups. **(D)** Quantification of Molecular markers of senescence induced by doxorubicin by RT-PCR in 4T1. A significant increase of C12-FDG positive cells was observed on day 1, day 3, and day 5 compared to the control. Significant elevation in the senescence markers, *CDKN1A* and *IL6* after doxorubicin expression. **(E)** Representative microscopic images of untreated and doxorubicin-treated (72 h) E0771 cells stained with β-gal. **(F)** Representative flow cytometry charts of E0771 C_12_-FDG positive cells in the experimental groups. **(G)** Statistical quantification of E0771 C_12_-FDG positive cells in the experimental groups. **(H)** Quantification of Molecular markers of senescence induced by doxorubicin by RT-PCR in E0771. A significant increase of C_12_-FDG positive cells was observed on day 3 compared to controls. Significant upregulation in the senescence markers, *CDKN1A* and *IL6* after doxorubicin expression. Values represent means ± standard error of means (SEM) of three independent experiments. ns; non-significant, ***; p < 0.001, ****; p < 0.0001 based on one-way ANOVA followed by Tukey’s honest significance post hoc test.

We initially evaluated the induction of senescence using sublethal concentrations of doxorubicin based on tumor cell viability studies (Supplementary Figure S1). The development of TIS in 4T1 and E0771 cell lines was established based on Senescence-associated-β-galactosidase (SA-β-gal) staining at day 3, and C_12_-FDG labeling-based flow cytometry quantification on days 1, 3, and 5 following doxorubicin exposure. C_12_-FDG is a surrogate for the upregulation of SA-β-gal (Debacq-Chainiaux et al., 2009). Figures 2A,E show that doxorubicin increased the number of blue-stained cells in both cell lines. In the 4T1 cell line, the percentage of C_12_-FDG-labeled cells represented 84.9%, 89.7%, and 81.2% of the total doxorubicin-treated cells relative to untreated cells on day 1, 3, and 5, respectively (Figures 2B,C). In the E0771 cell line, the C_12_-FDG-labeled cell population increased on day 1 from 22.1% to 35.9% on day 3 following doxorubicin exposure (Figures 2F,G) while declining to 22.9% on day 5. The highest percentage of C_12_-FDG-stained cells was detected on day 3 (72 h) in both cell lines, indicating that peak TIS development occurs 72 h post-doxorubicin treatment. TIS development was further confirmed by increased expression of *CDKN1A,* which supports the induction of DNA damage-triggered cell cycle arrest in both 4T1 and E0771 cell lines (Figures 2D,H). Moreover, the 2 cell lines exhibited increased expression of the SASP-related gene *IL6* (Figures 2D,H).

### 3.2 Influence of venetoclax and navitoclax on doxorubicin-exposed 4T1 and E0771 cells

**FIGURE 3:**
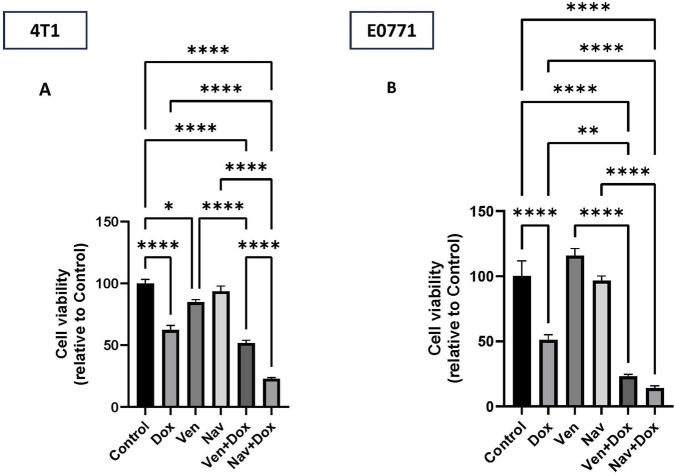
Navitoclax and venetoclax reduce cell viability in doxorubicin-induced senescent breast cancer cells. 4T1 and E0771 cells were treated with navitoclax and venetoclax, either alone or after senescence induction by doxorubicin. **(A)** Quantification of cell viability in the experimental groups in 4T1 using MTT assay. Navitoclax significantly inhibited cell growth after the induction of senescence by doxorubicin relative to doxorubicin treatment alone. **(B)** Quantification of MTT-based cell viability assay in E0771 cells. Venetoclax and navitoclax significantly reduced cell viability of doxorubicin-induced senescent cells. Values represent means ± standard error of means (SEM). *; p < 0.05, **; p < 0.01. ***; p < 0.001, ****; p < 0.0001 based on one-way ANOVA followed by Tukey’s honest significance post hoc test.

Next, the ability of the dual Bcl-2/Bcl-xL inhibitor, navitoclax, and the selective Bcl-2 inhibitor, venetoclax to reduce the viability of doxorubicin-induced senescent breast tumor cell lines after 24 h of treatment was assessed (Figure 3). None of the tested BH3 mimetics had a substantial impact on the tumor cells unless they had undergone prior exposure to doxorubicin (Figures 3A,B). Both navitoclax and venetoclax were effective in suppressing the growth/viability of E0771 cells induced into senescence by doxorubicin (Figure 3B). However, only navitoclax was effective against doxorubicin-induced (senescent) 4T1 cells, with only a minimal effect by venetoclax (i.e., the effect of venetoclax + doxorubicin was not significantly greater than for doxorubicin alone) (Figure 3A).

### 3.3 The combination of navitoclax and venetoclax with doxorubicin is associated with differential promotion of apoptosis

**FIGURE 4:**
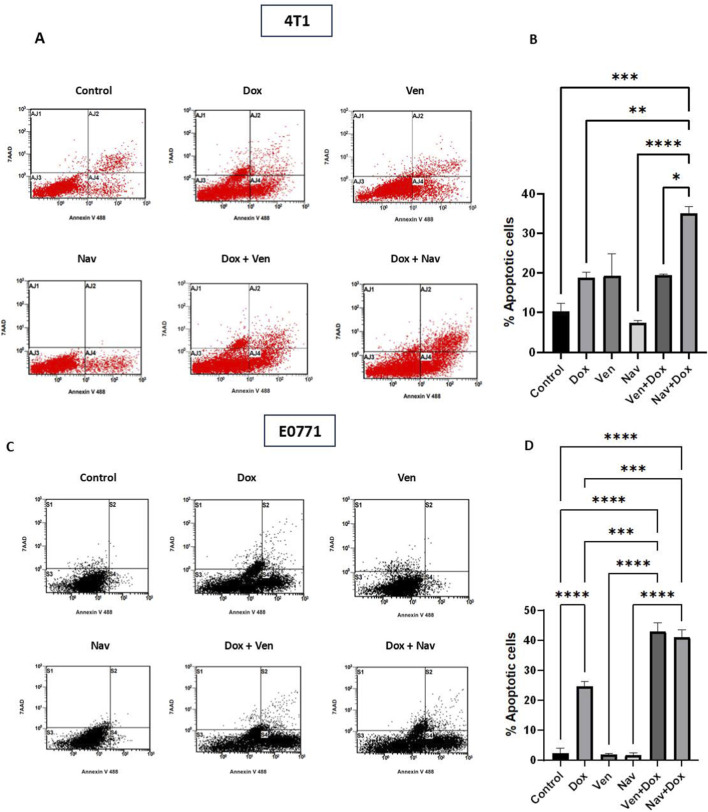
Navitoclax promotes apoptosis in doxorubicin-exposed cells more effectively than venetoclax. Apoptotic cells in the experimental groups were quantified using a flow cytometry-based assay. **(A)** Representative flow cytometry charts of apoptotic 4T1 cells stained with Annexin-V and 7-AAD. The x-axis represents the intensity of cells stained with Annexin-V that underwent apoptosis, while the Y-axis represents the intensity of cells stained with 7-AAD, which indicates nonviable cells. **(B)** Quantification of apoptotic populations in 4T1 cells under the indicated experimental conditions. Navitoclax significantly shifted cells to apoptosis after the induction of senescence by doxorubicin compared to doxorubicin treatment alone. **(C)** Representative flow cytometry charts of E0771 apoptotic cells in the experimental groups. **(D)** Quantification of apoptotic populations in the experimental groups in E0771. Venetoclax and navitoclax significantly increased the apoptotic subpopulations after the induction of senescence by doxorubicin relative to doxorubicin treatment alone. Values represent means ± standard error of means (SEM) of three independent experiments. *; p < 0.05, **; p < 0.01. ***; p < 0.001, ****; P < 0.0001 based on one-way ANOVA followed by Tukey’s honest significance post hoc test.

In previous studies relating to BH3 mimetics, their ability to enhance chemosensitivity has been associated with the promotion of apoptosis. A significant elevation in apoptotic cells was observed in the doxorubicin-treated group compared to the control group in E0771 cells (Figures 4B,D). Neither venetoclax nor navitoclax alone induced apoptosis in untreated E0771cells beyond control levels (Figures 4B,D). Representative flow cytometry charts of E0771 cells stained with Annexin-V and 7-AAD (Figure 4C) show increased apoptotic cell populations with the combination treatment relative to other experimental groups. When venetoclax or navitoclax was administered to doxorubicin-exposed breast tumor cells, there was a significant shift to apoptosis relative to doxorubicin (alone)-treated cells in E0771 cells (Figure 4D). Representative flow cytometry charts of 4T1 cells (Figure 4A) show a slight shift towards apoptosis in response to venetoclax treatment but not navitoclax alone. However, this shift was not statistically significant. Quantification of apoptotic cell populations (Figure 4B) revealed a significant increase in apoptosis in the doxorubicin-treated cells relative to untreated cells. Notably, navitoclax alone did not induce apoptosis in naïve 4T1 cells beyond the control cells. However, when navitoclax was applied to doxorubicin-exposed 4T1 cells, there was an approximately two-fold increase in the percentage of apoptotic cells relative to the doxorubicin-only group, indicating enhanced apoptosis with sequential navitoclax exposure (potentially post-senescence induction). However, increased apoptosis was not detected in the venetoclax/doxorubicin-treated groups (Figure 4B), which indicates the lack of sensitization of 4T1 cells by venetoclax.

### 3.4 The combination of navitoclax and venetoclax with doxorubicin is accompanied by reduced *CDKN1A* and *IL6* expression

**FIGURE 5:**
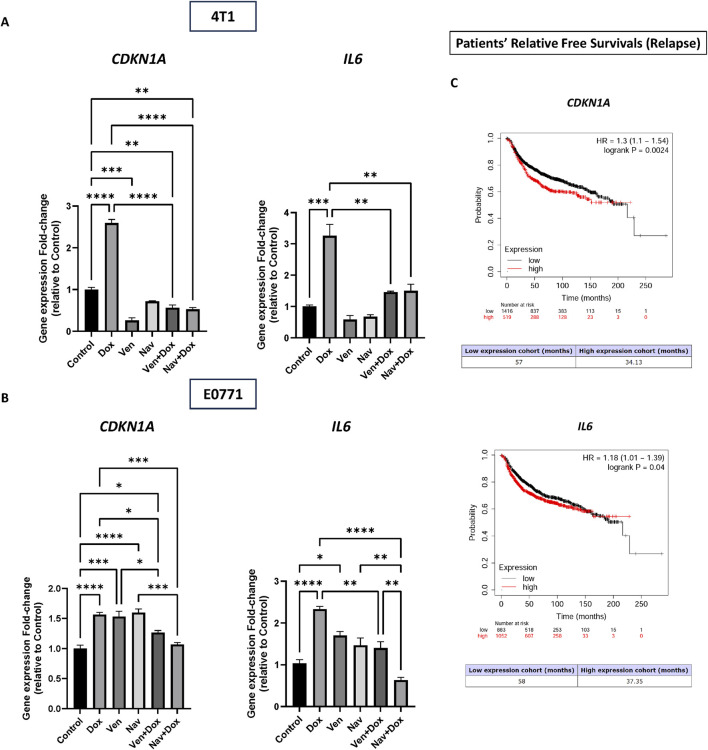
Reduced *CDKN1A* and *IL6* expression as a result of treatment by venetoclax and navitoclax. Total RNA was extracted from the experimental groups in 4T1 and E0771 cells. Gene expression was quantified using RT-PCR. **(A)** *CDKN1A* and *IL6* expression in the 4T1 cell line. The combination therapy groups significantly inhibited *CDKN1A* expression relative to cells treated with doxorubicin alone. *IL6* was significantly downregulated in the combination therapy groups in comparison to the doxorubicin alone group. **(B)** *CDKN1A* and *IL6* expression in the E0771 cell line. Doxorubicin followed by venetoclax or navitoclax demonstrated a significant downregulation of *CDKN1A* expression relative to doxorubicin-alone treated cells. *IL6* expression was significantly downregulated in only the combination therapy of the doxorubicin and navitoclax group in comparison to the doxorubicin alone group. Values represent means ± standard error of means (SEM) of three independent experiments. *; *p* < 0.05, **; *p* < 0.01. ***; *p* < 0.001, ****; *p* < 0.0001 based on one-way ANOVA followed by Tukey’s honest significance post hoc test. **(C)** Prognostic significance of *CDKN1A* and *IL6* expression profile (relative free survival) of breast cancer patients who underwent chemotherapy. Free survival probability was computed, and Kaplan-Meier curves were generated using kmpot.com. Patients with a low expression profile of *CDKN1A* were significantly less likely to relapse compared to patients with a low expression profile (HR = 1.3; P = 0.0024). The median relapse time was 57 months in the low-expression cohort, while the median relapse time was 34.13 months in the high-expression cohort. Patients with a low expression profile of *IL6* were significantly less likely to relapse compared to patients with a low expression profile (HR = 1.18; P = 0.04). The median relapse time was 58 months in the low-expression cohort, while the median relapse time was 37.35 months in the high-expression cohort. Kaplan-Meier curves from KMplotter database show high expression of *CDKN1A* and *IL6* correlates with reduced relapse-free survival in chemotherapy-treated breast cancer patients (n = 1935).

The effects of both venetoclax and navitoclax on senescence-related gene, *CDKN1A*, and SASP gene, *IL6*, following doxorubicin-induced senescence were assessed in both cell lines using RT-PCR (Figures 2,5). In 4T1 cells, treatment with 1 µM doxorubicin for 2 hours significantly upregulated *CDKN1A* expression by more than two-fold relative to untreated cells (*p* = 0.030). Venetoclax or navitoclax alone did not alter the expression level of *CDKN1A*. However, venetoclax or navitoclax treatment following doxorubicin-induced senescence resulted in a significant reduction in *CDKN1A* expression compared to cells treated with doxorubicin alone. Additionally, *IL6* expression showed a similar pattern, which was significantly upregulated after doxorubicin treatment. Neither BH3 mimetic altered the expression of *IL6* compared to the control. However, the combination therapy groups exhibited a significant downregulation of *CDKN1A* compared to the doxorubicin-treated group.

In E0771 cells, doxorubicin and navitoclax significantly upregulated *CDKN1A* relative to the untreated group. Exposure to venetoclax or navitoclax following the induction of senescence significantly inhibited *CDKN1A* expression in cells pulse-exposed to doxorubicin compared to doxorubicin alone. Furthermore, *IL6* expression was significantly increased upon doxorubicin treatment. *IL6* expression was downregulated in the combination-treated cells compared to the doxorubicin-exposed cells. However, the downregulation of *IL6* was more prominent in cells treated with navitoclax following the induction of senescence by doxorubicin relative to venetoclax. These results indicate that venetoclax and navitoclax treatment, when used against doxorubicin-exposed 4T1 cells, is associated with reduced *CDKN1A* and *IL6* expression, which could largely be due to a reduced burden of senescent tumor cells.

The impact of *CDKN1A* and *IL6* expression profiles on the relapse-free survival of breast cancer patients who underwent chemotherapy was investigated to assess the clinical significance of the association of these two genes with recurrence. Kaplan-Meier curves were generated using the KMplot.com tool. Figure 5C illustrates the association between the expression levels of *CDKN1A* and *IL6* and the relapse-free survival probability in these patients. Patients with high expression profiles of *CDKN1A* had a 30% higher likelihood of relapse compared to those with low expression profiles (*p* = 0.0024). Similarly, *IL6* is significantly associated with relapse (HR = 1.18; p = 0.04).

### 3.5 Both venetoclax and navitoclax suppressed growth of E0771 cells after doxorubicin treatment in immunocompetent animals

**FIGURE 6:**
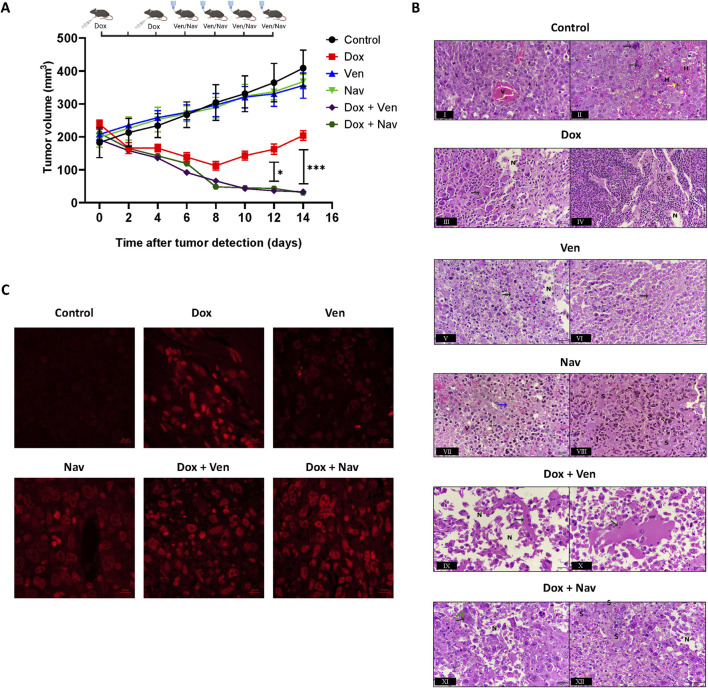
*In vivo* tumor growth is suppressed by navitoclax and venetoclax in combination with doxorubicin in C57BL/6 mice. **(A)** Tumor volume (mm^3^) quantification in the mouse experimental groups. 2,500,000 E0771 cells were implanted subcutaneously in C57BL/6 mice aged 6–8 weeks (n = 6 per group). Tumor volume was assessed every day until the volume reached around 200 mm^3^, when the mice were randomized into six groups: mice treated with drug-free vehicle (black), mice treated with two intraperitoneal (IP) doxorubicin doses of 2.5 mg/kg on day 0 and day 4 (red), mice treated with four oral venetoclax doses of 100 mg/kg on days, 6, 8, 10, and 12 after tumor detection (blue), mice treated with four oral navitoclax doses of 50 mg/kg on days, 6,8,10, and 12 after tumor detection (light green), mice treated with the combination therapy of doxorubicin and venetoclax based on the dose mentioned above protocol (purple), mice treated with the combination therapy of doxorubicin and navitoclax based on the dose mentioned above protocol (green). The tumor volume was significantly smaller in the mice treated with the combination therapy groups compared to those treated with doxorubicin alone on day 12 and day 14. Values represent means ± standard error of means (SEM) of each group (n = 6). Two-way ANOVA followed by Sadiac *post hoc* test was used. *; *p* < 0.05 and ***; *p* < 0.001 between Dox and Dox + Ven or Dox + Nav. **(B)** Representative photomicrographs of sectioned tumor nodules stained with hematoxylin and eosin, two of each treatment group. Sections were photographed using Nikon YS100 light microscope at ×400 magnification. Tumor tissues treated with the combination therapies exhibited increased necrotic areas (N) and inflammatory cells with stroma (S) relative to other experimental groups. Macrophages, hemosiderin granules, tumor cells with karyolitic nuclei, and multinucleated tumor cells were highlighted with black, yellow, blue, and green arrows, respectively. V; blood vessel, H; hemorrhage, N; necrotic area, S; stroma. **(C)** Representative TUNEL images of *in vivo* tumor tissue samples. The extent of apoptosis was quantified based on the intensity of the BrdU-Red signal. Tumor cells treated with doxorubicin followed by either venetoclax or navitoclax showed markedly higher BrdU-Red fluorescence intensity, indicating a significant induction of apoptosis, compared with the other experimental groups.

Based on the effectiveness of both venetoclax and navitoclax in combination with doxorubicin in the cell culture studies with E0771 cells, we evaluated the combination treatment against *in vivo* tumor growth following doxorubicin treatment in immunocompetent animals (C57BL/6 mice implanted with E0771 cells). Tumor volume quantifications are presented in Figure 6A. Two doses of doxorubicin administered on days 1 and 4 inhibited tumor growth for 8 days relative to untreated tumors. However, proliferation recovery of doxorubicin-treated tumors was observed starting on day 10. Four doses of either venetoclax or navitoclax alone, beginning on day 6, showed no significant effect on tumor growth compared to untreated tumors. However, both drugs dramatically reduced tumor volume following doxorubicin treatment, particularly on days 12 and 14. Unlike the findings from cell culture studies which shows an advantage of navitoclax in culling doxorubicin exposed (senescent) cancer cells, *in vivo* findings underscore the synergistic effect of both BH3 mimetics when used in conjunction with doxorubicin sequentially, enhancing anti-tumor efficacy beyond that observed with doxorubicin alone. These data show that venetoclax, like navitoclax, is capable of reducing tumor volume following doxorubicin treatment in immunocompetent mice.

### 3.6 Both venetoclax and navitoclax promoted *in vivo* tumor apoptosis and necrosis following doxorubicin treatment

Histological analysis of tumor sections stained with hematoxylin and eosin provided further insights into the cellular and morphological changes induced by the experimental treatments. Untreated subcutaneous tumor nodules displayed densely arranged rows of tumor cells with pleomorphic, relatively rounded nuclei and an open chromatin pattern, along with a rich blood supply (Figure 6BI). Minimal destruction was observed according to Evan’s grade, I (Table 1). Other sections showed the presence of giant macrophages, hemorrhage, and hemosiderin granules (Figure 6BII). Tumor nodules treated with doxorubicin showed loose tumor tissue, with cells changing from rounded with abundant nuclei to spindle-shaped with shrunken and elongated nuclei. The majority of cells appeared degenerated, leading to necrotic areas with increased stromal presence and the occurrence of giant macrophages for debris clearance (Figure 6BIII). Additionally, leukocytic infiltration was observed as a response to tumor degeneration (Figure 6BIV). Venetoclax promoted necrosis and inflammation in tumor cells (Figures 6BV,VI). Tumor nodules treated with navitoclax displayed tumor cells mixed with inflammatory cells and penetrated by blood vessels. Tumor nodules treated with navitoclax showed tumor cells close to each other, mixed with inflammatory cells, and penetrated with blood vessels. Most cells exhibited karyolitic nuclei (Figure 6BVII), while other sections revealed increased stromal depositions (Figure 6BVIII). All the monotherapy groups were ranked as IIa based on Evan’s grading (Table 1). The combination therapy groups, Doxorubicin plus either venetoclax or navitoclax, remarkably induced necrosis with a higher presence of inflammatory cells within the stroma (S) compared to other treatment groups, with a more notable necrotic effect of venetoclax (Figures 6BIV,X) relative to navitoclax (Figures 6BXI,XII). Such destruction was found significantly different relative to both control and doxorubicin-treated tissues, with the highest Evans’ grades, IIb, among all the experimental groups (Table 1).

**TABLE 1:** Immunohistochemical analyses of tumor tissues in E0771-bearing mice according to Evans’ grade.

Groups	Viable cells count	Destruction %	Evan’s grade
Control	6,319 ± 88	2	I
Dox	3,801 ± 131*a	40	IIa
Nav	5,505 ± 279*a,b	13	IIa
Ven	4,095 ± 317*a	35	IIa
Dox + Nav	2,631 ± 143*a,b	52	IIb
Dox + Ven	1,481 ± 184*a,b	63	IIb

Viable cell counts represented by the mean ± SEM, p ≤ 0.05 considered significant.

*a significant relative to control, *b significant relative to both control and doxorubicin-treated groups.

In accordance with the histopathological evaluation, tumor tissues were examined for apoptosis using the TUNEL assay. The microscopic images presented in Figure 6C demonstrate that doxorubicin-induced senescent tumor cells treated with either venetoclax or navitoclax exhibited clear evidence of apoptosis compared with the respective monotherapy groups. Untreated tumor cells displayed minimal fluorescence intensity, indicating an absence of apoptotic activity. These findings suggest that both venetoclax and navitoclax show greater capacity toward senescent tumor cells than toward naïve tumor cells.

## 4 Discussion

Although TIS can be considered as a favorable outcome in cancer treatment, wherein cancer cells cease to proliferate (Roninson, 2003; Ewald et al., 2010), emerging evidence suggests that senescence serves as a mechanism whereby tumor cells can survive chemotherapy (or radiation) and regain proliferative capacity (Milanovic et al., 2018; Saleh et al., 2019b; Duy et al., 2021; Xiao et al., 2023). Thus, the development of therapeutic strategies for eradicating senescent cancer cells has the potential to enhance treatment clinical outcomes. Senolytics have emerged as potential agents to selectively target senescent cells (Zhu et al., 2015; Kirkland and Tchkonia, 2017; Kirkland et al., 2017). In the context of cancer, navitoclax has demonstrated pronounced senolytic activity following senescence-inducing agents such as cisplatin (Ahmadinejad et al., 2022), temozolomide (Beltzig et al., 2022), etoposide (Wang et al., 2017), gemcitabine (Yamamoto et al., 2021; Yamamaoto et al., 2022), and androgen deprivation (Carpenter et al., 2021; 2024). In breast cancer specifically, navitoclax possesses a robust killing capacity against doxorubicin-induced senescent tumor cells *in vitro* and in immunodeficient mouse models *in vivo* (Saleh et al., 2020b; Shahbandi et al., 2020; de Paula et al., 2023). Other examples in the literature include studies by Estepa-Fernández et al. showing senolytic potential for navitoclax in palbociclib-induced senescent MDA-MB-231 breast tumors in nude (immunodeficient) BALB/c mice (Estepa-Fernández et al., 2023). In contrast, venetoclax has not previously shown a wide spectrum senolytic potential (Yosef et al., 2016). Nevertheless, venetoclax has shown promising results in several pre-clinical and clinical studies as monotherapy and in combination therapy, including with doxorubicin (Morschhauser et al., 2018; Lafontaine et al., 2021; As Sobeai et al., 2022), while the clinical implementation of navitoclax (especially for solid tumors) continues to face many challenges. Given that venetoclax is now approved standard-of-care in CLL and AML, investigation of its potential senolytic activity in solid tumor models appears to be essential. In this work, both navitoclax and venetoclax treatment after doxorubicin significantly suppressed the growth of breast cancer cells relative to doxorubicin treatment in E0771 cells in culture. As expected, both BH3 mimetics failed to suppress the proliferation of cancer cells that were not exposed to doxorubicin (potentially non-senescent).

Our results suggest that the sensitivity of breast tumor cells to navitoclax, and potentially venetoclax, is enhanced when the cells are induced into senescence, presenting an opportunity for dose optimization to mitigate unwanted toxicity, especially thrombocytopenia (Roberts et al., 2012; Kipps et al., 2015). Similar findings were previously reported *in vitro* in 4T1 cells, where navitoclax significantly, and in a dose-dependent fashion, reduced their viability following their induction into senescence by palbociclib (González-Gualda et al., 2020). However, it is important to acknowledge that our findings cannot definitively establish that the observed enhancement in growth suppression by both navitoclax or venetoclax is solely attributable to the culling of senescent cells. An alternative, and equally plausible interpretation, is that these two BH3 mimetics may instead be potentiating the cytotoxic effects of doxorubicin by lowering the apoptotic threshold, independent of a senolytic (senescence-selective) mechanism. Doxorubicin induces a complex cellular stress response that encompasses DNA damage, mitochondrial dysfunction, and activation of multiple pro-survival pathways, including BCL-2 family proteins (Takahashi, 2003; Schirone et al., 2022; Kciuk et al., 2023). Thus, the increased sensitivity we observed could reflect a synergistic interaction in which BH3 mimetics counteract compensatory anti-apoptotic defenses activated during doxorubicin exposure, thereby facilitating apoptosis even in cells that have not fully entered a stable senescent state. This model aligns with previous literature demonstrating that BCL-2, BCL-xL, and MCL-1 upregulation can serve as critical resistance mechanisms following genotoxic chemotherapy, and that their inhibition restores apoptotic priming without necessitating a senescent intermediate (Letai, 2008; Ni Chonghaile and Letai, 2008).

Although the capacity of venetoclax to eliminate doxorubicin-exposed tumor cells *in vitro* was not as pronounced as for navitoclax, both drugs demonstrated similar activity in the tumor bearing mice. The potential of venetoclax in doxorubicin-induced triple-negative breast cancer models was also recently reported by another research group (Schreiber et al., 2025). This work demonstrated that venetoclax can enhance doxorubicin-induced cytotoxicity regardless of p53 status of the tested breast cancer cell lines in culture, with increased apoptosis markers such as cleaved-PARP and -caspase-3 (Schreiber et al., 2025). While our studies support the findings of this report showing a potential of venetoclax in several breast cancer cell lines induced into senescence by doxorubicin, the Schreiber et al. studies appear to more likely to reflect an additive effect of both drugs in some of the tested cell lines, given that venetoclax alone significantly reduced cell viability based on the relatively high concentrations utilized (Schreiber et al., 2025). Moreover, venetoclax seemed to enhance cancer cell killing in cells exposed to doxorubicin but exhibiting minimal features of senescence, further supporting the proposal that our data might reflect a combinational synergistic effect rather than a senescence-selective senolytic effect of venetoclax (Schreiber et al., 2025). Similarly, a sarcoma cell model showed that venetoclax would reduce viability of senescent cells (induced by irradiation) at high concentrations (25 µM which is 2.5 fold higher than the highest concentration we used in this work); the impact of venetoclax on unirradiated, non-senescent counterparts, again suggested that the combination effects were largely additive (Lafontaine et al., 2021). Schwarzenbach et al. showed that the addition of venetoclax to senescent LN-229 cells (induced by temozolomide) would result in a dose-dependent enhancement of apoptosis; however, the effect of venetoclax on non-senescent cells was not presented (Schwarzenbach et al., 2021). Lastly, the senolytic potential of venetoclax *in vitro* seemed to be more evident when combined with other Bcl-2 family targeting agents such as Bcl-xL-specific degraders (Khan et al., 2024).

With respect to apoptosis, when sensitization to doxorubicibin by navitoclax was observed, there was a concomitant increase of apoptosis relative to doxorubicin-treated cells in both 4T1 and E0771 cells. However, venetoclax was able to significantly induce apoptosis only in doxorubicin-exposed E0771 tumor cells. It is noteworthy that although doxorubicin induced a relatively modest senescent fraction in E0771 cells (∼36% SA-β-gal–positive), subsequent exposure to venetoclax or navitoclax still produced a proportional ∼20% increase in apoptosis. This close correspondence between the magnitude of senescence induction and the extent of senolytic-triggered apoptosis supports, although not definitively, the interpretation that both agents are likely to have induced apoptosis in a senescence-selective manner in this model. These findings were validated in the *in vivo* experiments, where both senolytic agents markedly induced apoptosis in the groups pre-treated with doxorubicin, compared with the other treatment groups in E0771 tumor-bearing mice. This effect may be attributed to the inhibition of upregulated anti-apoptotic proteins, Bcl-2, Bcl-xL, and Bcl-W. This inhibition liberates pro-apoptotic proteins such as Bax and Bak, which ultimately promote the apoptosis cascade (Peña-Blanco and García-Sáez, 2018; Hauseman et al., 2020; Qian et al., 2022). It is noteworthy that sensitivity of senescent breast tumor cells to navitoclax can vary with low Noxa expression, rendering such cells resistant to apoptosis, where senolysis can only be achieved with concomitant Mcl-1 inhibition (Shahbandi et al., 2020; Al Shboul et al., 2023). In the work by Schreiber et al., the combination treatment led to mitochondrial outer membrane permeabilization, resulting in cytochrome c release and activation of the intrinsic apoptotic pathway which is a classical event that explains BH3 mimetic’s function (Schreiber et al., 2025). More importantly, the study reported that venetoclax disrupted the interaction between Bcl-2 and pro-apoptotic proteins, thereby sensitizing cells to doxorubicin-induced cell death.

In our study, both venetoclax and navitoclax also significantly reduced *CDKN1A* gene expression after doxorubicin exposure relative to doxorubicin or navitoclax monotherapy in both cell lines. These data suggest that the two drugs may manifest their activity, in part, through the suppression of p21^Cip1^ activity. It is worth mentioning that both cell lines were *TP53* mutant (Berglind et al., 2008; Le Naour et al., 2020; Schrörs et al., 2020). Therefore, our findings suggest that the role of *CDKN1A* in senescence is likely to be p53-independent. Alternatively, reduced expression of *CDKN1A* following senolytics treatment can also be attributed to the reduced burden of senescent tumor cells that were killed by apoptosis. This is likely to explain the observed gene expression results, and the role of senolytics in eliminating *CDKN1A* -mediated senescence. Our data also demonstrated a significant decrease in *IL6* expression in navitoclax-treated cells after TIS by doxorubicin. These results indicate that navitoclax successfully eradicated and reduced the burden of senescent cancer cells. Conversely, while 4T1 cells were sensitive to navitoclax, but not venetoclax, the latter was successful in reducing *CDKN1A* and *IL6* mRNA levels, which is unlikely then to be precipitated by reduced cell burden. Alternatively, venetoclax and related Bcl-2 inhibitors have been reported to exert anti-inflammatory and gene expression–modulatory effects independent of senescence clearance, which are often largely context dependent (Hernández-Silva et al., 2022; Wang et al., 2022; Jiang et al., 2024; Almudimeegh et al., 2025; Mani et al., 2025). These findings emphasize that *CDKN1A* and *IL6* may not serve as reliable indicators of senolytic activity across experimental models.

The most critical component of the current work is the finding that both venetoclax and navitoclax were capable of demonstrating senolytic potential in an immunocompetent mouse model. The majority of previous findings comparing the senolytic effects of navitoclax and venetoclax, or demonstrating the senolytic potential of venetoclax, were derived from studies conducted in immunodeficient animals. For example, the combination of doxorubicin with venetoclax demonstrated higher tumor growth inhibition in a model of immunodeficient mice challenged with MDA-MB-231 human breast tumor cells when compared to each agent alone (Inao et al., 2018; Schreiber et al., 2025). Interestingly, venetoclax had minimal, if any, effect on tumor growth in this model when used alone, unlike its concentration-dependent ability to kill non-doxorubicin-treated (non-senescent) breast cancer cells *in vitro* (Inao et al., 2018; Schreiber et al., 2025). Only when venetoclax was combined with doxorubicin was it able to exert a synergistic effect *in vivo,* which was coupled with a reduction in the senescent tumor cell burden (Schreiber et al., 2025). It is noteworthy that in these studies, doxorubicin and venetoclax were administered simultaneously, while we utilized a sequential approach of administering doxorubicin prior to navitoclax or venetoclax. In our work, both drugs delayed the observed recovery of tumor growth following the cessation of doxorubicin treatment *in vivo*. Only two other reports in the literature have presented similar observations using murine breast tumor cell lines and demonstrated equally robust results, confirming the premise for the “one-two punch” strategy for cancer treatment (González-Gualda et al., 2020; Shahbandi et al., 2020). However, these two reports were limited to testing the effect of only navitoclax. Our work represents the first attempt to demonstrate the *in vivo* senolytic potential for venetoclax in a breast cancer model in immunocompetent animals. We should also note that in this work, no direct evidence has been presented on doxorubicin-induced senescence *in vivo*, although this has been repeatedly shown in the literature. For instance, systemic administration of doxorubicin (5 mg/kg i.v., every 5 days × 3) increases SA-β-gal activity in PyMT breast cancer brain metastases, accompanied by loss of Lamin B1 and upregulation of additional senescence markers (Uceda-Castro et al., 2022). Other independent studies, including our own work in MDA-MB-231 xenografts, similarly report increased SA-β-gal staining in doxorubicin-treated tumors (Saleh et al., 2020b; Chaib et al., 2024; Xiang et al., 2024).

Although the present work supports the use of senolytics for breast cancer treatment in combination with standard of care therapies, several limitations must be acknowledged. First, navitoclax has been associated with adverse effects that have hindered its clinical approval. One approach involves dose optimization of navitoclax to facilitate its combination with other therapies, thereby reducing toxicity and enhancing the efficacy of anticancer drugs. It is also important to note that navitoclax can induce neutropenia, a condition that can be exacerbated by the concurrent administration of conventional chemotherapy (Puglisi et al., 2011). To address this issue, in this and previous studies, we have proposed the sequential administration of navitoclax following senescence-inducing chemotherapy, referred to as “sequential senolytic treatment”. This approach, which is equivalent to the “one-two” punch strategy first proposed by Rene Bernards (Wang et al., 2022), is intended to mitigate the potential toxicity of navitoclax. However, it is important to conduct toxicological studies to evaluate the safety of this sequential administration, particularly concerning its effects on platelets. Another, possibly more serious limitation to this work is that venetoclax was effective in combination with doxorubicin only in the E0771 cells. However, in the possible anticipation of the utilization of venetoclax in the clinic in solid tumors in combination with standard of care, it appears to be critical to define the tumor-associated factors that predict its effectiveness in preclinical studies. It is also possible that the enhanced *in vivo* efficacy of venetoclax compared to its *in vitro* activity may reflect immune-mediated mechanisms not captured *in vitro* or in immunodeficient models. To our knowledge, this is among the first studies to evaluate venetoclax as a senolytic in immunocompetent animals.

## 5 Conclusion

Our study investigated the combinational effects of venetoclax and navitoclax with doxorubicin in two breast cancer cell lines. Our findings demonstrated that venetoclax and navitoclax can demonstrate differential combinational activity in a preclinical model of triple negative breast cancer, *in vitro* and *in vivo*, potentially targeting doxorubicin-treated tumor cells and promoting apoptotic cell death. However, these outcomes are not consistent across the different cell lines studied, and the factors that confer susceptibility to this combinational strategy remain to be more fully defined. Our study suggests that further analysis is required prior to considering BH3 mimetics for breast cancer treatment as senolytics but encourages their use for potentially delaying senescence-mediated disease recurrence.

## Data Availability

The raw data supporting the conclusions of this article will be made available by the authors, without undue reservation.

## Supplementary material

SUPPLEMENTARY FIGURE S1 Optimization of doxorubicin concentration for senescence induction. 4T1 and E0771 cell lines were treated with several concentrations of doxorubicin ranging from 01 to 10 µM for 24 h. Cell viability was quantified at the end of the experiment using the MTT assay. 4T1 cells were more sensitive to doxorubicin treatment (IC50 = 0.772 µM) relative to E0771 cells (IC50 = 3.403 µM).
