# Supplementary figures and images for: Differential responses to the combination of navitoclax and venetoclax with doxorubicin in murine models of triple negative breast cancer
Source: Front Cell Dev Biol. 2026 Feb 5;14:1661424. doi: 10.3389/fcell.2026.1661424 (PMC12916633; doi:10.3389/fcell.2026.1661424)

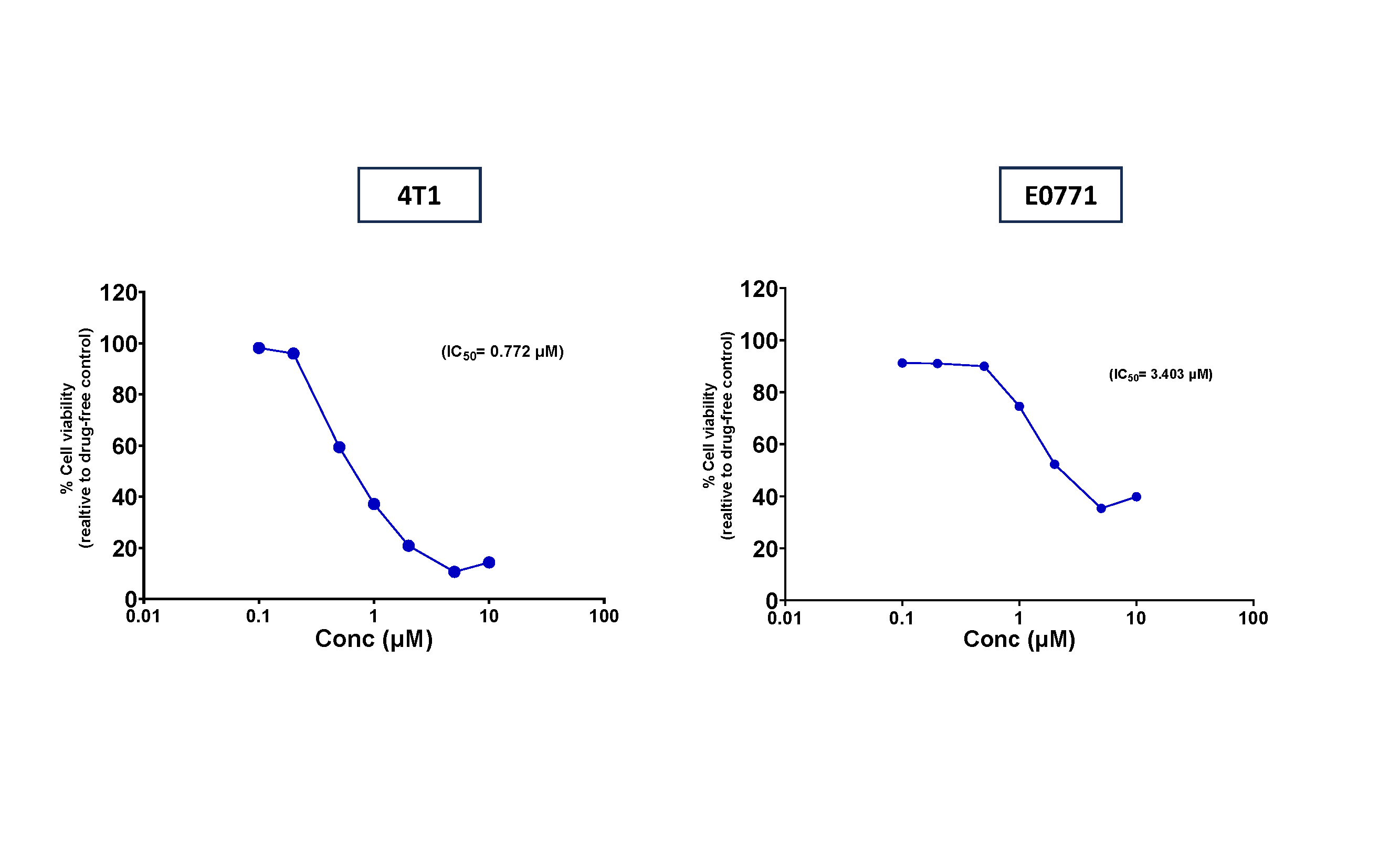

Supplement: Supplementary file 1 [file Image1.tif]
